# Flying under the radar: figurative language impairments in focal lesion patients

**DOI:** 10.3389/fnhum.2014.00871

**Published:** 2014-11-03

**Authors:** Geena R. Ianni, Eileen R. Cardillo, Marguerite McQuire, Anjan Chatterjee

**Affiliations:** ^1^Section on Neurocircuitry, Laboratory of Brain and Cognition, National Institute of Mental Health, National Institutes of HealthBethesda, MD, USA; ^2^Department of Neurology, Center for Cognitive Neuroscience, University of PennsylvaniaPhiladelphia, PA, USA

**Keywords:** metaphor, aphasia, focal lesion patients, figurative language, case study, sentence comprehension

## Abstract

Despite the prevalent and natural use of metaphor in everyday language, the neural basis of this powerful communication device remains poorly understood. Early studies of brain-injured patients suggested the right hemisphere plays a critical role in metaphor comprehension, but more recent patient and neuroimaging studies do not consistently support this hypothesis. One explanation for this discrepancy is the challenge in designing optimal tasks for brain-injured populations. As traditional aphasia assessments do not assess figurative language comprehension, we designed a new metaphor comprehension task to consider whether impaired metaphor processing is missed by standard clinical assessments. Stimuli consisted of 60 pairs of moderately familiar metaphors and closely matched literal sentences. Sentences were presented visually in a randomized order, followed by four adjective-noun answer choices (target + three foil types). Participants were instructed to select the phrase that best matched the meaning of the sentence. We report the performance of three focal lesion patients and a group of 12 healthy, older controls. Controls performed near ceiling in both conditions, with slightly more accurate performance on literal than metaphoric sentences. While the Western Aphasia Battery (Kertesz, 1982) and the objects and actions naming battery (Druks and Masterson, 2000) indicated minimal to no language difficulty, our metaphor comprehension task indicated three different profiles of metaphor comprehension impairment in the patients’ performance. Single case statistics revealed comparable impairment on metaphoric and literal sentences, disproportionately greater impairment on metaphors than literal sentences, and selective impairment on metaphors. We conclude our task reveals that patients can have selective metaphor comprehension deficits. These deficits are not captured by traditional neuropsychological language assessments, suggesting overlooked communication difficulties.

## INTRODUCTION

Metaphor is pervasive in everyday language, and often used to communicate complex, abstract, or unfamiliar concepts. Individuals encounter metaphors on a daily basis in the classroom *(The Bohr model atom is a tiny solar system*), in their social lives *(Our first date was a train wreck*), and in the media *(Congress froze the budget*). As a communication device, metaphor is practical, allowing familiar information to sculpt and inform new concepts. Conceptualized this way, metaphor is fundamental to the flexibility of human thought, revealing novel commonalities, facilitating learning, and enabling abstraction (Lakoff and Johnson, 1980; Gentner, 1983).

Despite the ubiquity of metaphor in thought and language, its neural instantiation remains uncertain. In an early formal demonstration of metaphor deficits following brain injury, Winner and Gardner (1977) found that right-hemisphere damaged (RHD) patients, but not left-hemisphere damaged (LHD) patients or healthy controls, had difficulty matching metaphoric sentences to pictures, suggesting the right hemisphere was uniquely tuned for metaphor comprehension. Several subsequent patient studies supported this claim (Brownell et al., 1984, 1990; Van Lancker and Kempler, 1987; Mackenzie et al., 1999; Champagne et al., 2004; Klepousniotou and Baum, 2005a,b). However, in some of these cases only RHD patients and controls were tested, providing no means of comparison between the hemispheres (Mackenzie et al., 1999; Champagne et al., 2004, 2007; Rinaldi et al., 2004) or RHD patients who performed at ceiling were excluded from analyses (Brownell et al., 1990). These studies sometimes also contained few items (e.g., as few as three or four in Brownell et al., 1990; Tompkins, 1990; Giora et al., 2000; Zaidel et al., 2002), showed that impairment depended on task (Winner and Gardner, 1977), or failed to show any hemispheric differences when task demands were accounted for statistically (Zaidel et al., 2002). Nonetheless, the first neuroimaging study of metaphor comprehension supported the right-hemisphere hypothesis (Bottini et al., 1994), bolstering the tentative claims made by the patient studies. Thus, the prevailing view became that metaphor comprehension was a lateralized, right hemisphere dominant process.

Many subsequent neuroimaging studies of metaphor comprehension, however, have failed to find the right-lateralized activations predicted by the right-hemisphere hypothesis of metaphor comprehension. Most studies report activation in both hemispheres (Eviatar and Just, 2006; Stringaris et al., 2006, 2007; Ahrens et al., 2007; Mashal et al., 2007; Chen et al., 2008; Bambini et al., 2011; Desai et al., 2011; Diaz et al., 2011; Cardillo et al., 2012; Lacey et al., 2012; Shibata et al., 2012; Uchiyama et al., 2012) and some only left-lateralized activations (Rapp et al., 2004, 2007; Lee and Dapretto, 2006; Kircher et al., 2007; Shibata et al., 2007; Mashal et al., 2009; Yang et al., 2009; Diaz and Hogstrom, 2011; Forgács et al., 2012). Recent meta-analyses confirm left-hemisphere dominance for figurative language, including metaphor. Although the right hemisphere is indeed often responsive to metaphoric stimuli, its contribution is neither equivalent to nor stronger than that of the left hemisphere; it is weaker (Rapp et al., 2012) or absent (Bohrn et al., 2012). Consistent with this conclusion, some patient studies found metaphor comprehension to be comparably impaired following left or right hemisphere injury (Tompkins, 1990; Gagnon et al., 2003), or more impaired following left than right injury (Giora et al., 2000).

Unsurprisingly, divergent lesion and neuroimaging data have not led to consensus regarding the laterality of metaphor comprehension (Schmidt et al., 2010). One explanation for these discrepancies is heterogeneity of stimuli and/or task demands. We have addressed stimulus design extensively elsewhere (Cardillo et al., 2010) and will address choice of task here. Tasks common in neuroimaging studies with healthy adults do not always extend well to patient populations. On the one hand, passive tasks like silent reading or periodic comprehension probes provide insufficient behavioral correlates for measurement. On the other hand, more demanding, semantic tasks like valence or plausibility judgment may elicit poor performance because of difficulty with the decision aspect of the task or a response-bias, not because of a comprehension problem, *per se*. These tasks also cannot tell us anything about what a person understood the sentence to mean. Comprehension of metaphoric sentences could be assessed with yes/no questions (Gagnon et al., 2003; Eviatar and Just, 2006; Prat et al., 2012), however, this task produces a relatively insensitive measure. Random guessing alone would produce 50% accuracy. Further, poor performance can only indicate a patient has metaphor comprehension difficulty, but provides no insight into the many possible reasons for a comprehension failure.

Experimental tasks commonly used with patients also present interpretive challenges. Evaluating metaphor comprehension with picture-matching may introduce visuospatial confounds in RHD patients, who perform better than LHD patients when asked to provide oral explanations of the same metaphors (Winner and Gardner, 1977; Mackenzie et al., 1999; Giora et al., 2000; Zaidel et al., 2002; Rinaldi et al., 2004). Oral explanations provide rich information but are difficult to quantify and necessitate fewer items than forced choice tasks (Giora et al., 2000; Zaidel et al., 2002; Champagne et al., 2004). In addition, some LHD aphasics may have difficulty conveying full comprehension in this format because of language production problems (Winner and Gardner, 1977). Semantic similarity judgments – in which a patient matches a metaphoric expression (e.g., *bright*) to its figurative sense (e.g., *clever*) – avoid many of the previously mentioned confounds. However, stimuli used in such tasks have been highly heterogeneous. Single words, dyads, and triads have all been used and studies have varied in how thoroughly or comparably they have matched answer choices and conditions on lexical confounds that are not of interest (Brownell et al., 1984, 1990; Gagnon et al., 2003).

Clinical assessments of language function following brain injury are even less discerning. Neurologists, speech pathologists, and neuropsychologists rely on diagnostic batteries to reveal compromised language skills, target speech-language rehabilitation approaches, and alert patients and their caregivers to areas of potential communication difficulty. The commonly administered Western Aphasia Battery (WAB; Kertesz, 1982), for instance, assesses spoken and written language production and comprehension, classifying patients by aphasia diagnosis and severity of impairment in different domains.

Although widely used, the WAB exclusively assesses literal language skills. Other aphasia assessments are similarly lacking. The Boston Diagnostic Aphasia Examination (Goodglass and Kaplan, 1983), the Porch Index of Communicative Ability (Porch, 1971), Minnesota Test for Differential Diagnosis of Aphasia (Schuell, 1965), and the Aphasia Diagnostic Profiles (Helm-Estabrooks, 1992) also do not contain any assessment or mention of metaphor. This clinical oversight runs contrary to common experience. Other batteries such as the Right Hemisphere Language Battery (Bryan, 1989) and Montreal Evaluation of Communications (Joanette et al., 2004) do include a figurative subtest but rely on items not motivated by current theoretical and methodological considerations relevant to metaphor comprehension (Cardillo et al., 2010; Schmidt et al., 2010). Furthermore, these batteries are rarely administered to patients with left hemisphere injury.

Given the limitations of existing metaphor comprehension tasks, we developed a new sentence-level, multiple-choice matching task to address these methodological challenges. Sentence stimuli – a staple of neuroimaging studies of metaphor – are preferable to single words, as they are metaphor’s most commonly encountered form. Their complexity however, requires careful balancing between figurative and literal conditions in terms of difficulty, a level of control that is rarely documented. Despite their naturalness and the feasibility of generating closely matched stimuli (e.g., Cardillo et al., 2010), sentence-level metaphors have not to our knowledge been used with patients. In our task, participants read a sentence and then chose from an array of four phrases the one that best matches its meaning (one correct target, three incorrect foils). This task has several advantages over other measures: (1) it avoids the visuospatial confounds of picture-matching, (2) it avoids the qualitative nature of oral explanations, (3) it avoids the low sensitivity of yes/no questions, (4) it uses naturalistic language, and (5) it explicitly acknowledges different metaphor subtypes. We demonstrate that the metaphor multiple choice task can be used to reveal unrecognized metaphor deficits in brain-injured patients by presenting three illustrative cases. We further demonstrate that this approach can identify metaphor-specific deficits, distinct from general comprehension deficits and unrecognized by traditional neuropsychological assessments of language. Finally, we show that systematically designed foils provide information about the nature of a patient’s comprehension failure.

## MATERIALS AND METHODS

### SUBJECTS

**FIGURE 1:**
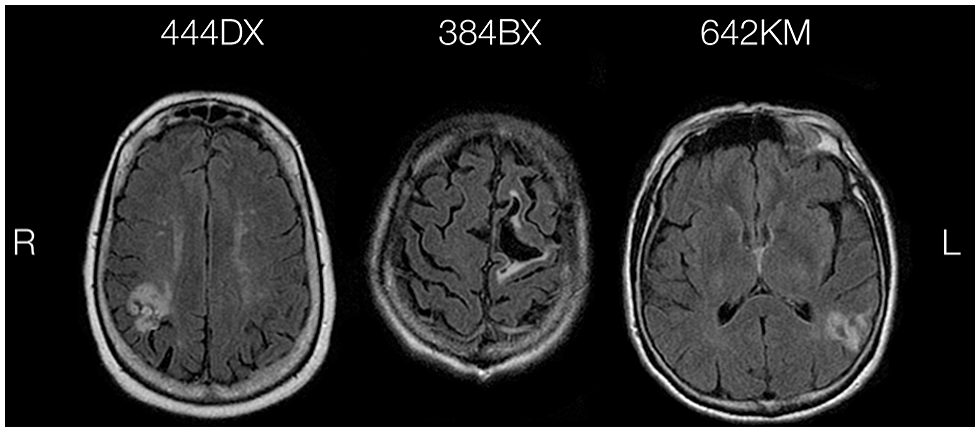
**Representative view of brain injury location in eachcase**.

Participants were three unilateral focal lesion patients enrolled in the University of Pennsylvania Focal Lesion Database. Patients with a history of other neurological disorders, psychiatric disorders, or substance abuse are excluded from the database. The patients presented here were drawn from an ongoing, large-scale group study of metaphor comprehension and specifically selected based on their observed behavioral patterns on our task. Sample size was dictated by the number of unique comprehension profiles that, when presented together, illustrate the capability of our task to detect and distinguish different kinds of metaphor impairment. Detailed demographic and neuropsychological information about the patients is provided in **Table 1** and an axial view of their injury location is provided in **Figure 1**.

**Table 1:** Demographic and neuropsychological profiles of cases.

Patient	Sex	Age	Education (years)	Lesion side	Region	Lesion volume^1^	Type of stroke	Chronicity (months)	P-BAC					WAB (AQ)^2^	OANB	
									Exec(26 max)	Mem(27 max)	VisSp(18 max)	Lang (12 max)	Beh(24 max)		Actions	Objects
444DX	F	81	12	R	PT	15496	Ischemic	120	21.5	15	13	11.5	24	95.5	94.0	93.0
384BX	M	74	12	L	F	11306	Hemorrhagic	143	19.5	14	13	10	24	91.3	100.0	98.8
642KM	M	78	12	L	P	7996	Ischemic	130	19	16	18	11	24	96.8	94.0	98.0

*T, temporal; P, parietal; F, frontal; Exec, executive function; Mem, Verbal/visual episodic memory; VisSp, visuospatial/visuoconstructional operations; Lang, lexical retrieval/language; Beh, behavior/social comportment.*

*^1^Voxel size = 1 mm × 1 mm × 1 mm; ^2^Within normal limits cut-off = 93.8.*

Patient 444DX is an 81 year-old retired factory worker who suffered an ischemic stroke 120 months prior to testing. The Philadelphia Brief Assessment of Cognition (PBAC), a brief dementia-screening instrument, was administered to assess function in five cognitive domains: working memory/executive control, lexical retrieval/language, visuospatial/visuoconstructional operations, verbal/visual episodic memory, and behavior/social comportment (Libon et al., 2011). Performance indicated compromised visuospatial, memory, and executive functions but normal language and social skills. Object and action naming battery (OANB) scores confirmed clinically normal lexical access for common object and action names (Druks and Masterson, 2000) and administration of the Western Aphasia Battery (Kertesz, 1982) likewise indicated clinically normal language abilities. An MRI scan demonstrated a lesion damaging the posterior temporal and parietal cortex of the right hemisphere.

Patient 384BX is a 74 year-old, retired butcher who suffered a hemorrhagic stroke 144 months prior to testing. Performance on the PBAC indicated compromised visuospatial, memory, and executive functions but normal language and social skills. Following injury he reported halting speech and stuttering. Administration of the WAB revealed some residual difficulty with naming and a diagnosis of mild anomia. OANB scores, however, indicated clinically normal lexical access for common object and action names. An MRI scan demonstrated a lesion undercutting the superior frontal gyrus of the left hemisphere.

Patient 642KM is a 78 year-old retired construction manager who suffered an ischemic stroke 130 months prior to testing. Performance on the PBAC indicated compromised memory and executive function but normal visuospatial, language, and social skills. OANB scores indicated clinically normal lexical access for common object and action names, and the WAB score indicated clinically normal language abilities. An MRI scan demonstrated a lesion damaging the parietal cortex of the left hemisphere.

Twelve neurologically healthy older adults recruited from the University of Pennsylvania Control Database served as a control population (Age: 64.3 ± 9.9, Education: 14.4 ± 2.6) and were paid $15/h for their participation. All participants were native English speakers, right-handed and gave informed consent to participate in accordance with the Institutional Review Board of the University of Pennsylvania.

### STIMULI

#### Sentences

Stimuli consisted of 60 metaphor-literal sentence pairs of three types. One third of the items were of the nominal-entity form, one third were of the nominal-event form, and one third were of predicate form. Nouns referring to concrete entities or objects (e.g., *bullet*, *cheetah, drum*) served as the metaphorical words in nominal-entity sentences, nominalized verbs in nominal-event sentences [e.g., *(a) dance, (a) limp, (a) fall*], and verbs in predicate sentences (e.g., *ran, giggled, argued*). All nominal-entity and nominal-event metaphors were of the form “*The X was a Y*” where *Y* was the word being used metaphorically. All predicate metaphors consisted of a noun phrase and an action verb followed by a prepositional phrase. In these items the verb was the word used metaphorically. It remains to be seen if different types of metaphor are also delineated at the cognitive or neural level (Cardillo et al., 2012). Given that objects and actions, as well as nouns and verbs, have been shown to differ in their semantic properties and neural instantiations (Damasio and Tranel, 1993; Martin et al., 1995; Kable et al., 2002, 2005) it is possible that their figurative extensions do as well. Although investigating the role of syntactic form and semantic properties of source terms was not the focus of this study, the possibility of encountering category-specific deficits dictated that different types of metaphor were balanced.

Forty nominal-entity, 40 nominal-event, and 40 predicate sentence pairs were selected from a superset of 624 sentence pairs [80 pairs were taken from Cardillo et al. (2010) and 80 pairs were drawn from a pool of 312 items designed and normed using identical methods] using Stochastic Optimization of Stimuli software (Armstrong et al., 2012). Optimized selection ensured metaphors and literals were matched in terms of familiarity, length (number of words, number of content words, number of characters), average content word frequency, average content word concreteness, and positive valence ratio (*p*’s > 0.10). As previously observed (Cardillo et al., 2010), metaphors were judged to be significantly less imageable (*p* < 0.005) and natural (*p* < 0.01) than their literal counterparts, and significantly more figurative (*p* < 0.005). Sentences of different types (nominal-entity, nominal-event, predicate) were further matched on interpretability (metaphors only), figurativeness (metaphors only), familiarity, naturalness, imageability, length (number of words, number of content words, number of characters), frequency, concreteness, and positive valence ratio (*p*’s > 0.10). Means and standard deviations of 12 collected psycholinguistic variables are summarized below in **Table 2**.

**Table 2:** Psycholinguistic properties of literal and metaphoric sentences.

	Literal			Metaphor		
	Nominal-Entity	Nominal-Event	Predicate	Nominal-Entity	Nominal-Event	Predicate
	
	*M (SD)*	*M (SD)*	*M (SD)*	*M (SD)*	*M (SD)*	*M (SD)*
Base auditory imagery	2.63 (1.2)	2.61 (1.4)	2.07 (1.16)	2.63 (1.2)	2.61 (1.4)	2.07 (1.16)
Base visual imagery	3.66 (1.14)	3.2 (0.59)	3.41 (0.72)	3.66 (1.14)	3.2 (0.59)	3.41 (0.72)
Concreteness	480 (76)	474 (46)	500 (53)	450 (57)	449 (69)	474 (76)
Frequency*	92.9 (159)	89.9 (142.4)	86.7 (85.3)	90.8 (123.7)	91.8 (128)	95.6 (133.7)
No. of characters	33.3 (4.2)	32 (5.1)	33.6 (5.2)	34.3 (4.6)	32.7 (5.2)	34.9 (4)
No. of words	6.1 (0.4)	6.2 (0.4)	6.2 (0.5)	6.1 (0.6)	6.1 (0.5)	6 (0.6)
No. of content words	3.2 (0.5)	3.2 (0.4)	3.3 (0.5)	3.2 (0.5)	3.1 (0.5)	3.3 (0.4)
Interpretability	n/a	n/a	n/a	0.94 (0.08)	0.94 (0.08)	0.96 (0.05)
Familiarity	5.28 (0.73)	5.14 (1.11)	5.26 (1.23)	4.96 (0.76)	4.83 (1.18)	4.86 (1.37)
Naturalness	5.68 (0.73)	5.76 (0.95)	5.48 (1.24)	4.84 (0.82)	5.1 (1.07)	4.8 (1.34)
Imageability	5.55 (0.83)	5.67 (0.97)	5.8 (1.08)	4.17 (0.97)	4.27 (0.78)	3.94 (1.16)
Figurativeness	1.88 (0.73)	2.02 (0.92)	1.78 (0.91)	5.62 (0.56)	5.28 (0.77)	5.25 (1.02)
Valence RT	1279 (213)	1390 (182)	1426 (237)	1351 (131)	1432 (220)	1495 (200)

*SUBTL_WF_ values from Brysbaert and New (2009).

#### Answer choices

Four answer choices were generated to accompany each sentence: one correct target and three incorrect foils. All answer choices were composed of an adjective or adverb, followed by a noun. As shown in **Table 3**, in the metaphor condition the target was related to the figurative meaning of the sentence, Foil 1 was related to the literal sense of the sentence, Foil 2 was the opposite of the metaphorical sense of the sentence, and Foil 3 was unrelated. Foils were designed to be informative of the type of language deficit present. A Foil 1 selection indicates a literal bias in metaphor comprehension. A Foil 2 selection indicates a semantic integration impairment, as the metaphorical sense of the source word was necessarily activated but incorrectly interpreted in the context of the sentence. A Foil 3 selection indicates a more general comprehension deficit, as it is entirely unrelated to the sentence.

**Table 3:** Sentence and answer choice examples.

Sentence	Syntax	Example	Target	Foil 1	Foil 2	Foil 3
Metaphor	Nominal-Entity	The coffee was a caffeine bullet.	energy jolt	military ammunition	soothing lullaby	funny teacher
	Nominal-Event	His interest was a mere sniff.	weak enthusiasm	runny nose	delighted fascination	rotten fruit
	Predicate	The debate spun into a brawl.	violent incident	twirling form	peaceful resolution	toxic fumes
Literal	Nominal-Entity	The police evidence was a bullet.	lethal weapon	confiscated goods	hospital bandage	circus tent
	Nominal-Event	The rabbit’s twitch was a sniff.	nose wiggle	epileptic fit	completely motionless	yoga class
	Predicate	The top spun into the box.	whirling motion	glass marble	fixed position	tiny sailboat

In the literal condition, the foils were designed to mirror the difficulty and nature of foil types in the metaphor condition as closely as possible. The target was related to the literal meaning of the sentence, Foil 1 was related to the agent of the sentence by category membership (but not implied by the sentence), Foil 2 was the opposite of the literal sense of the sentence, and Foil 3 was unrelated. It was necessarily impossible to make Foil 1 answers of the same nature as Foil 1 answers in the metaphor condition, but by presenting a strong lexical associate of one of the content words, Foil 1 answers were designed to mirror the semantic selection demands of Foil 1 answers in the metaphor condition (which presented a meaning strongly associated with the source term). Given the reversed valence necessarily entailed by the Foil 2 condition (the opposite of the target meaning), an additional constraint on all answer choices was introduced to avoid valence-related biases in selection: for both metaphor and literal items, Target and Foil 2 had opposite valences and Target and Foil 3 had the same valence.

Finally, frequency values for the answer choices were collected from SUBTLEXus (Brysbaert and New, 2009). No significant differences in average frequency were found between literal and metaphor conditions, between sentence types, or between answer choices. Concreteness values were also collected from the MRC Psycholinguistic Database (Coltheart, 1981) and the University of South Florida Norms (Nelson et al., 2004). For those words that did not have published concreteness values, we collected our own using the procedures of Cardillo et al. (2010). Given the abstract nature of metaphor, Target and Foil 1 answer choices were significantly different in terms of average concreteness (*p* < 0.005). In order to avoid any concreteness-related bias in selection, an additional constraint on all answer choices was introduced: Target and Foil 3 also significantly differed in concreteness (*p* < 0.005) and the target and Foil 2 did not (*p* > 0.10). Literal answer choices also followed this pattern: Target and Foil 1 differed in concreteness (*p* < 0.001), as did Target and Foil 3 (*p* < 0.005), but Target and Foil 2 did not (*p* > 0.10). As such, answer choices were matched on frequency, concreteness and valence so none could aid blind guessing. **Table 3** provides examples of sentence and answer choice stimuli. Full materials are available upon request.

### PROCEDURE

#### Control procedure

All participants made judgments on all 120 items. Subjects were told to choose the single answer choice which best matched the “meaning of the sentence,” and to guess if unsure. The task was self-paced. Participants pushed the space bar once for the sentence to appear. After reading the sentence for comprehension, participants pushed the space bar again to view the answer choices. Answer choices were presented in quadrant format below the sentence, Participants were instructed to indicate an answer choice using four keys on the keyboard. Sentences were presented centrally in black, 18-point font on a white background using E-Prime 1.1 software on a Dell Inspiron laptop. Each participant received a unique, random order of items. The target and each foil had a 25% chance of appearing in any single quadrant on the screen in any given trial. Ten practice trials preceded four blocks of experimental trials.

#### Patient procedure

The patients’ task was similar to the controls’ with one modification: the trials were advanced by the experimenter. The experimenter pressed the spacebar for the sentence to appear. This was followed by a 3 s delay, and then the answer choices were presented beneath the sentence. To avoid motor response and memory difficulties, patients indicated an answer by pointing to or saying the answer aloud and the experimenter recorded this answer using the keyboard.

### BEHAVIORAL ANALYSIS

An item analysis of healthy controls’ scores revealed three items whose comprehension fell 3 SD below the average; these items were eliminated from further analysis. A subject analysis of accuracy scores revealed a single individual whose comprehension fell 3 SD below average on any given sentence-type; this individual was replaced. For controls, accuracy for literal and metaphor conditions was averaged across all participants. For patients, accuracy in the literal and metaphor conditions was calculated separately for each individual. Foil profiles were generated for each patient by dividing the number of each type of error (Foil 1, Foil 2, Foil 3) by the total number of errors in literal and metaphor conditions.

We tested for a comprehension deficit in the metaphor condition at the level of the individual patient using “Bayesian analysis for a *simple* difference,” developed by Crawford et al. (2010). The analysis was done on standardized scores and repeated for the literal condition. This test uses Bayesian Monte Carlo methods to determine if a patient’s score is sufficiently below the scores of controls such that the null hypothesis, that the patient’s score is an observation from the control population, can be rejected. In this case, patients with a *simple* metaphor or literal deficit exhibit significantly reduced comprehension in that condition, relative to controls.

We also tested for a differential deficit in metaphor comprehension at the level of the individual patient using “Bayesian analysis for a *differential* difference,” developed by Crawford et al. (2010). The Bayesian test for a *simple* difference can only indicate whether a patient is impaired in the metaphor, literal, or both conditions. It does not distinguish between reduced accuracy due to difficulty with metaphor specifically and reduced accuracy due to a general impairment affecting literal and metaphoric language alike. The Bayesian test for a *differential* difference however, can make this distinction by also taking into account the differential accuracy score and correlation between the two conditions, as established by the control group. Patients with a *differential* metaphor deficit exhibit proportionally greater difficulty with metaphoric than literal sentences than is observed in the control population.

## RESULTS

Overall, the control group performed near ceiling. Literal accuracy (*M* = 96.8, SD = 1.98) was significantly higher than metaphor accuracy (*M* = 93.5, SD = 4.65); *t*(11) = 2.744; *p* = 0.019). The correlation between literal and metaphor accuracy was *R* = 0.516 (*p* = 0.044). In the metaphor condition, Foil 1 (the literal sense of the sentence), was the most common error (66.7%), followed by Foil 2 (24.4%) and Foil 3 (8.9%). In the literal condition, Foil 1 (related to the agent of the sentence by category membership, but not implied by the sentence), was the most common error (78.3%), followed by Foil 2 (17.4%) and Foil 3 (4.3%).

### GENERAL SENTENCE COMPREHENSION IMPAIRMENT (444DX)

Application of the Bayesian test for a simple deficit revealed a simple metaphor comprehension deficit [*t*(11) = -3.653; *p* < 0.01] and a simple literal comprehension deficit [*t*(11) = -5.004; *p* < 0.001], in 444DX. Application of the Bayesian test for a differential deficit revealed a non-significant difference in metaphor and literal comprehension scores, indicating a general sentence comprehension impairment. 444DX made predominantly Foil 1 and Foil 2 errors in both the metaphor and literal conditions. See **Table 4** for detailed reporting of single case statistics.

**Table 4:** Single case statistics and foil profile of 444DX.

	Control sample				Patient														
					Single bayes				Differential bayes							Foil Profile			
					Case Scores	Significance Test	Estimated % of control population obtaining lower score than case		Estimated effect size		Significance Test	Estimated % of control population obtaining discrepancy more extreme than case		Estimated effect size					
	n	Mean(%)	SD(%)	SE(%)	444DX (%)	*p*	Point(%)	95% CI lower limit (%)	Point	95% CI lower limit	*p*	Point(%)	95% CI lower limit (%)	Point	95% CI lower limit	Errors	Foil 1(%)	Foil 2 (%)	Foil 3 (%)
Literal	12	96.8	1.98	0.63	86.4	0.0001871	0.02	0.00	-5.253	-7.077	0.17082	17.08	0.02	-1.487	-3.609	8	25	75	0.0
Metaphor	12	93.5	4.65	1.47	75.9	0.0019561	0.20	0.00	-3.785	-5.135						14	64.3	28.6	7.1

### DISPROPORTIONATE IMPAIRMENT IN METAPHOR COMPREHENSION (384BX)

Application of the Bayesian test for a simple deficit revealed a simple metaphor comprehension deficit [*t*(11) = -8.640; *p* < 0.005] and a simple literal comprehension deficit [*t*(11) = -4.182; *p* < 0.001] in 384BX. Application of the Bayesian test for a differential deficit revealed a differential metaphor deficit [*t*(11) = 4.656; *p* < 0.02]. In the metaphor condition, 384BX’s errors were overwhelmingly Foil 1, while Foil 2 accounted for the majority of errors in the literal condition. See **Table 5** for detailed reporting of single case statistics.

**Table 5:** Single case statistics and foil profile of 384BX.

	Control sample				Patient														
					Single bayes				Differential bayes							Foil Profile			
					Case Scores	Significance Test	Estimated % of control population obtaining lower score than case		Estimated effect size		Significance Test	Estimated % of control population obtaining discrepancy more extreme than case		Estimated effect size					
	n	Mean(%)	SD(%)	SE(%)	444DX (%)	*p*	Point(%)	95% CI lower limit (%)	Point	95% CI lower limit	*p*	Point(%)	95% CI lower limit (%)	Point	95% CI lower limit	Errors	Foil 1(%)	Foil 2 (%)	Foil 3 (%)
Literal	12	96.8	1.98	0.63	88.1	0.0007162	0.07	0.00	-4.394	-5.94	0.01309	1.31	0.00	4.656	1.575	7	14.3	85.7	0.0
Metaphor	12	93.5	4.65	1.47	51.7	0.0000016	0.00	0.00	-8.989	-12.052						28	92.9	7.1	0.0

### SELECTIVE IMPAIRMENT IN METAPHOR COMPREHENSION (642KM)

Application of the Bayesian test for a simple deficit revealed a simple metaphor comprehension deficit [*t*(11) = -5.790; *p* < 0.0001] in 642KM. Literal comprehension was not significantly different than that of controls. Application of the Bayesian test for a differential deficit revealed a differential metaphor deficit [*t*(11) = 5.129; *p* < 0.001]. Like 444DX, 642KM made predominantly Foil 1 and Foil 2 errors in both the metaphor and literal conditions. See **Table 6** for detailed reporting of single case statistics.

**Table 6:** Single case statistics and foil profile of 642KM.

	Control sample				Patient														
					Single bayes				Differential bayes							Foil Profile			
					Case Scores	Significance Test	Estimated % of control population obtaining lower score than case		Estimated effect size		Significance Test	Estimated % of control population obtaining discrepancy more extreme than case		Estimated effect size					
	n	Mean(%)	SD(%)	SE(%)	444DX (%)	*p*	Point(%)	95% CI lower limit (%)	Point	95% CI lower limit	*p*	Point(%)	95% CI lower limit (%)	Point	95% CI lower limit	Errors	Foil 1(%)	Foil 2 (%)	Foil 3 (%)
Literal	12	96.8	1.98	0.63	94.9	0.1882557	18.83	6.42	-0.96	-1.52	0.00065	0.06	0.00	5.129	2.966	3	33.3	66.7	0.0
Metaphor	12	93.5	4.65	1.47	65.5	0.0000610	0.01	0.00	-6.022	-8.099						20	55.0	35.0	10.0

To summarize, the three patients exhibited three distinct deficit patterns. 444DX demonstrated general sentence level impairment; she was impaired on both metaphor and literal comprehension, but not significantly more so on either condition. 384BX demonstrated a disproportionate metaphor deficit; he was impaired on both metaphor and literal comprehension, but significantly more so for metaphors. 642KM demonstrated a selective metaphor deficit; he was impaired on metaphors but displayed normal literal comprehension.

## DISCUSSION

Metaphors are powerful and pervasive communication devices in everyday language, yet conspicuously absent from standard clinical assessments of language. The purpose of this study was to demonstrate that a metaphor multiple-choice task can reveal profiles of impaired metaphor comprehension in brain-injured patients that go undetected by traditional aphasia assessments. Three unilateral focal lesion patients made judgments on 60 matched literal-metaphor sentence pairs by choosing the phrase that best matched the meaning of a given sentence from an array of four possible answers. Compared to a group of healthy, older adults, single-case statistics revealed three unique patterns of impaired metaphor comprehension in the three patients (444DX, 384BX, 642KM). None of these patterns were predicted by their performance on standard clinical measures of receptive and expressive language.

Although the WAB is widely used to diagnose and classify aphasia following brain injury, it is agnostic with respect to figurative language, including metaphor. Our data indicate profound, unrecognized deficits in this domain, impairments that can persist post-injury despite normal literal language comprehension, and may significantly impact daily communication and thinking. All three cases in our series were impaired in their comprehension of metaphoric sentences, but the specific pattern of performance suggests these deficits were of three different natures.

444DX was impaired in both literal and metaphoric conditions. The absence of a differential deficit suggests that her difficulty with metaphor reflects a general sentence comprehension impairment. 444DX’s low performance is surprising considering her near perfect accuracy on the WAB, OANB, the language subsection of the PBAC, and casual conversation. One possibility is that her behavior reflects, at least in part, difficulty with the semantic executive demands of the task. A multiple choice problem requires the systematic consideration and rejection of competing meanings before selecting the correct one. 444DX’s performance on the PBAC indicated impaired memory and executive function, domain general deficits would reasonably impact strategic processing in the linguistic domain as well. Consistent with a difficulty in resolving semantic competition, 444DX remarked, “Some of them were tricky. A lot of times, I thought there were two correct answers. I doubted myself several times.”

384BX was also significantly impaired in both his literal and metaphoric comprehension, responding correctly to only 88% of the literal sentences, and only 52% of the metaphoric sentences. Unlike 444DX, however, the difference between his metaphoric and literal comprehension was greater than would be expected in healthy adults, indicating a disproportionate difficulty with metaphor. This pattern suggests that a milder, lexical-semantic comprehension impairment is present in addition to a metaphor-specific deficit. The severity of 384BX’s diagnosed anomia, however, is mild and not suggestive of the severe metaphor impairment observed. Furthermore, anomia is classified as an expressive aphasia, in which language production is affected while comprehension is relatively preserved. Therefore 384BX’s poor metaphor comprehension cannot be anticipated by the anomia diagnosis. Nor is he aware of his difficulty. In debriefing he remarked, “I started stuttering after the stroke,” but “I can still read and remember,” and “I did not feel like my reading was affected (by the injury).”

Most dramatic was the disproportionate metaphor deficit demonstrated by 642KM. Consistent with his high scores on the neuropsychological tests and conversational ease, his performance in the literal condition was near ceiling – yet he responded correctly to only 66% of metaphoric sentences. This pattern indicates his comprehension failure is specific to metaphor and cannot be explained by general language comprehension problems. Like 384BX, 642KM remained unaware of his impairment even after testing, remarking, “it was easy,” and “I understood ninety percent of what I was reading.” As these comments suggest, this comprehension problem is not only unrecognized by traditional aphasia assessments, but is also opaque to the patient himself.

As the three cases illustrate, not all metaphor deficits are alike. Some deficits are “pure,” selective for metaphor while leaving literal language intact (642KM). In other patients this metaphor-specific deficit is accompanied by a milder comprehension deficit affecting literal language as well (384BX). Still other metaphor-deficits are reflective of a general deficit, impacting metaphoric and literal language comprehension similarly (444DX). The close matching of metaphoric and literal conditions on psycholinguistic variables enables confident direct comparison of metaphor and literal comprehension. By contrast, many previous studies have tested patients on only metaphoric items (Winner and Gardner, 1977;

Mackenzie et al., 1999; Giora et al., 2000; Zaidel et al., 2002; Champagne et al., 2004; Rinaldi et al., 2004), designs that cannot preclude the possibility of a general comprehension deficit, rather than a metaphor-specific one.

The unique foil profiles of each patient further illustrate the diversity of metaphor deficits. 384BX’s errors in the metaphor condition were overwhelmingly Foil 1 (literal interpretation). This pattern indicates his metaphor comprehension fails in a specific way, resulting in a systematic, highly implausible misinterpretation. Literal biases have been reported previously in brain-damaged patients by Brownell et al. (1984) and Rinaldi et al. (2004), using picture-matching and a single-word semantic similarity judgment task, respectively. The present study is the first demonstration of literal bias for metaphor comprehension in which metaphor and literal items were closely matched on average and in pairwise fashion. Thus, we may confidently attribute comprehension deficits to difficulty with metaphors, rather than potentially confounding sentence properties (e.g., familiarity, length, frequency, concreteness, etc.). In contrast to 384BX, 642KM, and 444DX showed more mixed foil profiles, with Foil 2 errors in addition to Foil 1 errors. Foil 2 errors indicate the metaphorical meaning was at least partially accessed, but incorrectly interpreted. This error pattern suggests that the origin of comprehension failure in cases like 444DX and 642KM is more complex than for patients presenting only a systematic literal bias. Understanding the different ways metaphor comprehension breaks down in the injured brain may enable more appropriate and targeted rehabilitation strategies.

Metaphor deficits are of clinical interest to patients and their caregivers for many of the same reasons as general language impairments, but their effects on communication may be more insidious. For example, metaphor is an attractive option for discussing internal emotional states (*I exploded at the rude customer*), abstract concepts (*The right thing to do is a gray area*) or explaining new, complex ideas (*The brain is a computer*). In these cases, a literal bias would make comprehending the metaphoric statements as they were intended impossible. Yet, as the normal neuropsychological profiles and the patients’ own reflections make plain, metaphor interpretation failures do not announce themselves immediately the way literal comprehension deficits do. The abstract nature of the concepts typically expressed by metaphor may contribute to their poor detection in casual conversation. More simply, we are imperfect listeners; if we expect successful comprehension, we are more likely to project it.

Finally, it is worth noting that both patients demonstrating a disproportionate metaphor deficit had unilateral left-hemisphere lesions (384BX, 642KM). Without overstating the importance of lesion location in such a small sample, this observation is inconsistent with the right-hemisphere hypothesis of metaphor, which predicts metaphor impairments in right- not left-hemisphere patients. In accordance with the accumulating evidence from neuroimaging, our data indicate metaphor comprehension is a not solely a right-hemisphere dependent process. Left-hemisphere brain-damaged patients may be in as much need for figurative language rehabilitation as right hemisphere injured patients. Research on the efficacy of therapies targeting metaphor comprehension is not only scarce, but also customarily only targets right-hemisphere patients because of their presumed susceptibility to these kinds of deficits (Lundgren et al., 2006, 2011).

In sum, our results from three illustrative patient cases establish the utility of a carefully designed multiple choice task as a new tool in the investigation of the neural basis of metaphor comprehension. Focal lesion patients were the focus of this investigation, but the approach is equally suitable for investigating questions of metaphor comprehension in other clinical populations or in neuroimaging studies with healthy adults. The metaphor multiple choice task uniquely avoids the methodological and interpretative pitfalls of tasks previously used with patients, while adding increased sensitivity for capturing different types of comprehension deficits. Further, although not the aim of the current study, the inclusion of metaphors of different types enables investigating current, outstanding theoretical questions about the cognitive and neural mechanisms supporting metaphor comprehension. Most importantly, we wish to highlight the clinical utility of our approach. Our task revealed that patients can have figurative language deficits neither evaluated nor predicted by traditional aphasia assessments. This observation raises the possibility that many patients that might benefit from targeted therapies are currently overlooked. We can not see what our tools are not designed to detect.

## AUTHOR CONTRIBUTIONS

The experiment was conceived by Eileen R. Cardillo and Anjan Chatterjee The stimuli were generated by Eileen R. Cardillo. The experiments were programmed and carried out by Geena R. Ianni and Marguerite McQuire Data analysis was done by Geena R. Ianni with assistance from Eileen R. Cardillo and Marguerite McQuire All authors were involved in data interpretation. The paper was written by Geena R. Ianni and revised by Eileen R. Cardillo, Marguerite McQuire and Anjan Chatterjee All authors approved the final version for submission.

## Conflict of Interest Statement

The authors declare that the research was conducted in the absence of any commercial or financial relationships that could be construed as a potential conflict of interest.
